# 3D Bioprinting of a Bioactive Composite Scaffold for Cell Delivery in Periodontal Tissue Regeneration

**DOI:** 10.3390/biom13071062

**Published:** 2023-06-30

**Authors:** Guohou Miao, Liyu Liang, Wenzhi Li, Chaoyang Ma, Yuqian Pan, Hongling Zhao, Qing Zhang, Yin Xiao, Xuechao Yang

**Affiliations:** 1 Guangzhou Key Laboratory of Basic and Applied Research of Oral Regenerative Medicine, Affiliated Stomatology Hospital of Guangzhou Medical University, Guangdong Engineering Research Center of Oral Restoration and Reconstruction, Guangzhou 510182, China; 2 School and Hospital of Stomatology, Guangzhou Medical University, Guangzhou 510182, China; 3 Hospital of Stomatology, Zhongshan 528404, China; 4 Laboratory for Myology, Department of Human Movement Sciences, Faculty of Behavioral and Movement Sciences, Vrije Universiteit Amsterdam, Amsterdam Movement Sciences, 1081 BT Amsterdam, The Netherlands; 5 School of Medicine and Dentistry, Menzies Health Institute Queensland, Griffith University, Gold Coast, QLD 4222, Australia; 6 The Australia-China Centre for Tissue Engineering and Regenerative Medicine (ACCTERM), Queensland University of Technology, Brisbane, QLD 4000, Australia

**Keywords:** 3D bioprinting, cell-laden scaffold, composite hydrogel, periodontal regeneration

## Abstract

Hydrogels have been widely applied to the fabrication of tissue engineering scaffolds via three-dimensional (3D) bioprinting because of their extracellular matrix-like properties, capacity for living cell encapsulation, and shapeable customization depending on the defect shape. However, the current hydrogel scaffolds show limited regeneration activity, especially in the application of periodontal tissue regeneration. In this study, we attempted to develop a novel multi-component hydrogel that possesses good biological activity, can wrap living cells for 3D bioprinting and can regenerate periodontal soft and hard tissue. The multi-component hydrogel consisted of gelatin methacryloyl (GelMA), sodium alginate (SA) and bioactive glass microsphere (BGM), which was first processed into hydrogel scaffolds by cell-free 3D printing to evaluate its printability and in vitro biological performances. The cell-free 3D-printed scaffolds showed uniform porous structures and good swelling capability. The BGM-loaded scaffold exhibited good biocompatibility, enhanced osteogenic differentiation, apatite formation abilities and desired mechanical strength. The composite hydrogel was further applied as a bio-ink to load with mouse bone marrow mesenchymal stem cells (mBMSCs) and growth factors (BMP2 and PDGF) for the fabrication of a scaffold for periodontal tissue regeneration. The cell wrapped in the hydrogel still maintained good cellular vitality after 3D bioprinting and showed enhanced osteogenic differentiation and soft tissue repair capabilities in BMP2- and PDGF-loaded scaffolds. It was noted that after transplantation of the cell- and growth factor-laden scaffolds in Beagle dog periodontal defects, significant regeneration of gingival tissue, periodontal ligament, and alveolar bone was detected. Importantly, a reconstructed periodontal structure was established in the treatment group eight weeks post-transplantation of the scaffolds containing the cell and growth factors. In conclusion, we developed a bioactive composite bio-ink for the fabrication of scaffolds applicable for the reconstruction and regeneration of periodontal tissue defects.

## 1. Introduction

Periodontal disease often results in defects in both bone tissue and soft tissue of gingiva [1,2,3,4,5]. However, current clinical treatments are unable to effectively repair both types of tissue simultaneously [6,7]. In recent years, the application of 3D printing technology in tissue engineering has provided the possibility for accurate adjustment of the spatial structure and pore structure of scaffolds [8,9,10]. In addition, 3D bioprinting technology has gradually become a research hotspot in the field of regenerative medicine because it can realize the layer-by-layer printing of biological inks containing biological materials, bioactive factors and cells, which provides a novel method for the personalized repair of tissue defects [11,12,13], especially for periodontal tissue defects.

The application of biomaterials in the treatment of periodontal tissue defects has been widely studied and has achieved good clinical achievements. For example, Muhammad S Shaikh et al. carried out meta-analyses regarding the clinical outcomes of nano-hydroxyapatite or bovine-derived hydroxyapatite/cell-binding peptide complexes for regeneration of periodontal defects; the results showed that the above two biomaterials showed better therapeutic effects than open-flap debridement in the treatment of periodontal intrabony defects [14,15]. However, this class of inorganic biomaterials is mostly granular and cannot be easily shaped to scaffolds for irregular bone defects. Hydrogels are considered as ideal scaffold materials for tissue repair due to their microstructures and physicochemical properties similar to the natural extracellular matrix (ECM), which is not only conducive to cell attachment, migration and proliferation [16,17], but also can encapsulate living cells and maintain cell phenotypes and induce differentiation [18]. Moreover, the cell-laden hydrogels are suitable as bio-inks for 3D bioprinting to construct personalized scaffolds and show great potential in diverse biomedical applications [19,20], particularly in periodontal tissue regeneration and repair. While a range of materials and techniques have been employed to design and construct cell-laden hydrogels for this purpose, there remains room for improvement in terms of in vitro bioactivity and the ability to regenerate both soft and hard periodontal tissue.

Gelatin methacryloyl (GelMA) is a kind of modified gelatin with photosensitive functional groups; it has attracted increasing interest in the preparation of 3D-printed bone tissue engineering scaffolds and cell delivery in recent years [21,22,23]. However, due to the poor mechanical properties and temperature sensitivity, it is difficult to directly print biological scaffolds by extrusion-based 3D printing, which restricts its use in bone tissue engineering. Blending of other biomaterials (sodium alginate, SA) capable of forming hydrogels has been used to improve the printability of bio-inks, the biological and mechanical properties of which can be custom-tailored according to different requirements [24,25,26]. Additionally, inorganic bioactive components (hydroxyapatite, nano clay, bioactive glass, etc.) are usually introduced to organic matrices to further enhance the biological properties of bio-inks [27,28]. Due to its excellent in vitro bioactivity, osteogenic activity and soft tissue repair ability, bioactive glass microspheres (BGMs) have attracted extensive attention in the field of tissue regeneration and have been used as bioactive fillers incorporated into hydrogel matrixes to improve their ability to repair bone or skin soft tissue [29]. The addition of BGM into GelMA hydrogels could significantly improve their physicochemical and biological properties [28]. Although GelMA, BGM and SA are promising materials for hydrogel scaffolds, their suitability as bio-inks for the 3D bioprinting of cell- and growth factor-loaded hydrogel scaffolds for periodontal tissue regeneration has yet to be determined.

**Figure 1:**
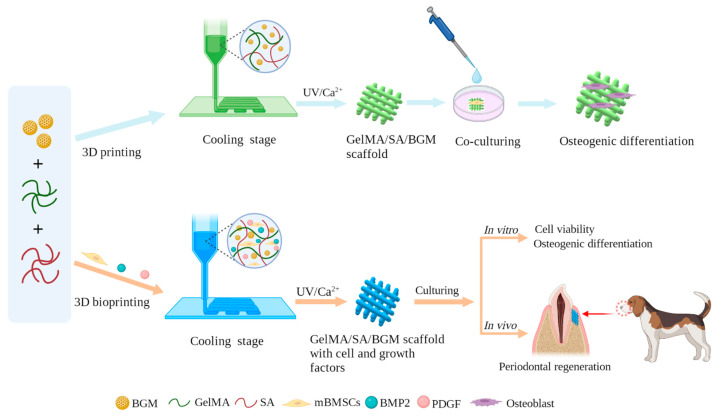
Flowchart of fabrication and characterization of 3D-printed GelMA/SA/BGM scaffold and 3D-bioprinted cell- and growth factor-laden GelMA/SA/BGM scaffold.

Therefore, in this study, we aimed to develop a novel composite hydrogel with good bioactivity and biological properties, which can be fabricated into porous scaffolds by 3D printing and can be used as a bio-ink for cell bioprinting to prepare personalized restorative scaffolds for regeneration of periodontal soft and hard tissue. GelMA, SA and BGM were employed to prepare a composite hydrogel and fabricated porous scaffolds by 3D printing. The physicochemical properties and in vitro biological performance of the 3D-printed scaffolds were investigated. After that, we applied the above-mentioned hydrogel as a bio-ink to living cells and growth factors for the fabrication of a scaffold for periodontal tissue. Mouse bone marrow mesenchymal stem cells (mBMSCs) were used as model cells wrapped in the 3D-bioprinted hydrogel scaffolds because of the multidirectional differentiation potential of stem cells. To further improve the periodontal tissue repair ability of the cell-loaded scaffolds, BMP-2 and PDGF were added to the hydrogel for promoting the simultaneous repair of bone tissue and gingival soft tissue. The survivability and differentiation capability of the cells in the scaffolds as well as the capability of in vivo periodontal tissue regeneration were studied. There are few reports regarding the construction of bifunctional cell-laden hydrogel scaffolds in combination with BMP2 and PDGF for periodontal tissue repair. This research will present a good option for 3D bioprinting of bioactive scaffolds for simultaneous repair of periodontal bone and soft tissue. The fabrication and characterization of 3D-printed GelMA/SA/BGM scaffolds and 3D-bioprinted GelMA/SA/BGM scaffolds with cell and growth-factor scaffolds are illustrated in Figure 1.

## 2. Materials and Methods

### 2.1. Materials

Anhydrous ethanol (AE), HCl, K_2_HPO_4_·3H_2_O, MgCl_2_·6H_2_O, Na_2_SO_4_ and NaCl were purchased from Sinopharm Chemical Reagent Co., Ltd. (Shanghai, China). Dodecylamine (DDA), Methacrylic anhydride (MA), Photoinitiator 2959 (PI-2959), Gelatin (G), sodium alginate (SA), CaCl_2_, Tetraethyl orthosilicate (TEOS), Triethyl phosphate (TEP) and Ca(NO_3_)_2_·4H_2_O (CN) were purchased from Aladdin Reagent Co., Ltd. (Shanghai, China). Collagenase type II, Tris, β-glycerophosphate sodium, Ascorbic acid, Dexamethasone and Cetylpyridinium chloride (CPC) were purchased from Sigma-Aldrich (Saint Louis, MO, USA). Dulbecco’s modified eagle’s medium (DMEM), Penicillin–Streptomycin Solution (100 U/mL streptomycin, 100 U/mL penicillin), fetal bovine serum (FBS), Phosphate buffer (PBS) and Trypsin-EDTA (0.25%) were purchased from Gibco (Thermo Fisher, Waltham, MA, USA). C57BL/6 mouse bone marrow mesenchymal stem cells (mBMSCs) were purchased from Cyagen Biosciences Inc. (Guangzhou, China). Triton X-100 was purchased from Beijing Dingguo Changsheng Biotechnology (Beijing, China). Cell Counting Kit-8 was purchased from Dojindo (Kumamoto Ken, Japan). BCIP/NBT Alkaline Phosphatase Color Development Kit was purchased from Beyotime Biotechnology (Shanghai, China). Alkaline Phosphatase testing kit (AKP testing kit) was purchased from Nanjing Jiancheng Biotechnology Co., Ltd. (Nanjing, China). Live-Death Staining Kit, BCA Protein Assay Kit and Alizarin Red staining solution (1%, pH 4.2) were purchased from Bestbio (Shanghai, China). RNase-free water was purchased from Leagene Biotechnology (Beijing, China). TRIzol™ Reagent was purchased from Invitrogen (Carlsbad, CA, USA). Prime Script^TM^ RT Master Mix (Perfect Real Time) and TB Green^TM^ Premix Ex Taq™ II (Tli RNaseh Plus) were purchased from Takara (Kyoto, Japan).

### 2.2. Synthesis of BGM

The BGM was synthesized according to the previous literature [30]. The molar composition of BGM was 60% SiO_2_, 36% CaO and 4% P_2_O_5_. Briefly, 3 g of DDA was dissolved in a mixed solution of AE (80 mL) and deionized water (25 mL) at 40 °C. Then, 10.9 mL of TEOS and 1.1 mL triethyl phosphate (TEP) were added successively at a rate of 0.5 mL/min, and each was stirred for 0.5 h. After that, 6.87 g of CN was added to the above solution, stirred for 3 h and aged for 24 h at room temperature. The white precipitates collected by centrifugation were washed alternately with AE and distilled water 2 to 3 times. The precipitates were dried in a freeze-dryer for 48 h and calcined at 600 °C for 3 h. The morphology and microstructure of BGM were characterized by scanning electron microscopy (SEM, DSM 982-Gemini, Zeiss, Oberkochen, Germany) and X-ray diffraction (XRD, X’pert PRO, PANalytical, Almelo, The Netherlands), respectively. The chemical composition was determined by energy dispersive spectroscopy (EDS).

### 2.3. Synthesis of GelMA

GelMA was prepared in accordance with a previous study [31]. Briefly, 10 g of G was fully dissolved into 100 mL of phosphate buffer saline (PBS) at 60 °C, and 8 mL of MA was slowly added at a rate of 0.5 mL·min^−1^ under vigorous stirring. After 3 h of reaction, the mixture solution was dialyzed against distilled water to remove unreacted components through dialysis membranes (12–14 kDa molecular weight cut-off) at 40 °C for 7 days. Finally, the dialyzed solution was centrifuged and freeze-dried, and the lyophilized GelMA was stored at −20 °C for future use.

### 2.4. Preparation of GelMA/SA and GelMA/SA/BGM 3D-Printed Scaffolds

Before printing, the inks were first prepared. The lyophilized GelMA (15% *w*/*v*), BGM (5% *w*/*v*) and PI 2959 (0.5% *w*/*v*) were dissolved in deionized water to obtain a homogenous ink. A certain amount of SA (10% *w*/*v*) solution was added to the above ink to improve its printability. The ink was transferred to a printing barrel with a nozzle (400 μm inner diameter) that was fixed to a 3D printer (3D-bio printer, Bio-Architect ^®^WS, Regenovo, Hangzhou, China). The distance between the grids was 1 mm. The temperature of the ink was set at 20 °C, and the printing platform was adjusted to 5 °C to make the extruded strands solidify and obtain an initial intensity. The GelMA/SA/BGM scaffold was printed as designed by extrusion, layer by layer. After printing, the scaffold was transferred to a 4% CaCl_2_ solution, cross-linked for 5 min, irradiated for 30 s under 365 nm UV light, washed with deionized water three times, frozen at −80 °C overnight, and finally freeze-dried for 48 h. Pure GelMA/SA scaffold without BGM was also prepared with the same parameters.

### 2.5. Characterization of 3D-Printed Scaffolds

#### 2.5.1. SEM Observation

The surface microstructure was characterized by scanning electron microscopy (SEM, S-3400N, Hitachi, Tokyo, Japan) at an accelerating voltage of 15 kV. Before observation, the lyophilized samples were sputter-coated with gold. The surface elemental composition was detected by an energy dispersive X-ray spectrometer (EDS) equipped on the SEM.

#### 2.5.2. Swelling Test

To measure the equilibrium swelling ratio, samples were completely lyophilized, and the dry weight was recorded (W_d_). Then, the samples were soaked in PBS at 37 °C for 24 h. The swollen samples were weighed (Ws) after gently wiping the surface using filter paper. The swelling ratio was calculated as (Ws − W_d_)/W_d_ × 100%.

#### 2.5.3. In Vitro Degradation Test

The degradation properties of composite hydrogel scaffolds were evaluated using collagenase solution. The lyophilized samples were weighed (W_0_) and immersed in collagenase II solution at a concentration of 2.5 U/mL at 37 °C. The samples were collected at specified time points (6, 12, 24, and 48 h), washed with deionized water, and then completely lyophilized. The weight of the dried samples after degradation was measured as W_d_. The degradation ratio was calculated with the following equation: (W_d_/W_0_) × 100%.

#### 2.5.4. Mechanical Properties

The compression properties of composite hydrogel scaffolds were measured by a universal testing machine (Instron 336, Norwood, MA, USA). The samples used in the compression test were prepared in a cylinder, 10 mm in diameter and 2 mm in height. The stress–strain curve was recorded at a compression rate of 0.5 mm. The compression modulus was calculated using the slope of the linear deformation stage (10–20%) of the stress–strain curve.

#### 2.5.5. In Vitro Bioactivity

The in vitro bioactivity of composite hydrogel scaffolds was evaluated by inducing apatite formation on the scaffold surface. All samples (10 mm × 10 mm × 5 mm) were immersed in 50 mL of 2 × simulated body fluid (SBF) at 37 °C. After 3 and 7 days of immersion, the samples were collected, freeze-dried, and observed by SEM and XRD.

### 2.6. In Vitro Biocompatibility

#### 2.6.1. Cell Viability and Morphology

Before cell experiments, the scaffolds were treated under UV irradiation and 75% ethanol immersion for sterilization, washed three times with PBS, placed in 12-well plates, and incubated with DMEM overnight. After the scaffolds were incubated in the cell incubator for 30 min, the cell suspension (1 mL, 1 × 10^6^ cells/mL) was added on each scaffold and incubated at 37 °C in 5% CO_2_. After 3 d and 7 d of culture, the cell viability on the scaffolds was evaluated using a Live-Death Staining Kit (Bestbio, Shanghai, China). The cell-laden scaffolds were rinsed with PBS three times and incubated with a 2 μM Calcein AM solution at room temperature for 30 min. Then, the scaffolds were thoroughly rinsed with PBS twice and incubated with 4 μM EthD-1 for 2 min. The living cells stained by Calcein AM and dead cells stained by EthD-1 were visualized using a fluorescence microscope (DMI3000B, Leica, Weztlar, Germany). SEM (S-3400N, Hitachi, Tokyo, Japan) was used to observe the cell morphology on the scaffolds after 4% paraformaldehyde fixation, gradient alcohol dehydration, and freeze-drying.

#### 2.6.2. Cell Proliferation

The scaffold extracts were used for cell proliferation and osteogenesis assays. Briefly, the sterilized GelMA/SA and GelMA/SA/BGM scaffolds were soaked in DMEM at a mass/volume ratio of 5 mg/mL to prepare the extracts. After 24 h of incubation, the supernatant was collected by centrifugation and sterilized using a 0.22 μm filter (Millipore). mBMSs were seeded into 96-well plates at a density of 1.5 × 10^3^ cells/well. After 24 h incubation, the culture medium was replaced with different extracts and incubated for 1, 3 and 7 days. The medium was changed every two days, and the DMEM without scaffolds immersion was used as the control group. At every time point, the culture medium was removed, and 200 μL of fresh medium containing 10% (*v*/*v*) CCK-8 solution was added and incubated for 2 h at 37 °C. A total of 100 μL of the medium was transferred to a new 96-well plate, and the absorbance was measured at 450 nm by a microplate spectrophotometer (Multiskan FC, Thermo, Waltham, MA, USA). Each group had three parallel samples.

### 2.7. The Osteogenic Activity of Composite Hydrogel Scaffolds

#### 2.7.1. Alkaline Phosphatase (ALP) Activity

mBMSCs were seeded in 48-well plates at a density of 1 × 10^4^ cells/well. After 24 h, the medium was replaced with GelMA/SA and GelMA/SA/BGM extracts containing osteogenic medium (10 nM dexamethasone, 10 mM β-glycerolphosphate and 50 ng/mL ascorbic acid). The osteogenic medium with extracts was used as the control group. The culture medium was changed every two days. At day 3 and 7, ALP staining was performed using a BCIP/NBT kit according to the manufacturer’s instruction. In addition, quantitative analysis of ALP was also detected by AKT assay kit cells according to the manufacturer’s instructions. The absorbance value was measured at 405 nm using 4-nitrophenol as the standard. The intracellular total protein content was determined by the BCA kit. Finally, the ALP activity was normalized to the total protein content. Each group had three parallel samples.

#### 2.7.2. Alizarin Red Staining and Accumulated Calcium Assay

mBMSCs were seeded in 48-well plates at a density of 1 × 10^4^ cells/well. After 24 h, the medium was replaced with GelMA/SA and GelMA/SA/BGM extracts containing osteogenic medium (10 nM dexamethasone, 10 mM β-glycerolphosphate and 50 ng/mL ascorbic acid). The osteogenic medium with extracts was used as the control group. The culture medium was changed every two days. After culturing for 14, 21 and 28 days, cells were rinsed with PBS, fixed, and incubated with 40 mM alizarin red S (ARS) (Sigma-Aldrich, Saint Louis, MO, USA) solution for 30 min to stain mineralized nodules. The stained nodules were observed using a stereoscopic microscope (EZ4HD, Leica, Weztlar, Germany). The quantitative analysis of ARS staining at day 21 was performed by adding 10% *w*/*v* cetylpyridinium chloride (Sigma-Aldrich, Saint Louis, MO, USA), and the absorbance of each well was detected at 562 nm.

#### 2.7.3. Osteogenesis-Related Gene Expression

mBMSCs were seeded in 6-well plates at a density of 1 × 10^5^ cells/well. After 24 h, the medium was replaced with GelMA/SA and GelMA/SA/BGM extracts containing osteogenic medium (10 nM dexamethasone, 10 mM β-glycerolphosphate and 50 ng/mL ascorbic acid). The osteogenic medium with extracts was used as the control group. The culture medium was changed every two days. After 3, 7 and 14 days, the expression of osteogenesis-related genes in mBMSCs, including alkaline phosphatase (ALP), aosteopontin (OPN), osteocalcin (OCN), runt-related transcription factor 2 (Runx2) and collagen typeⅠ(ColⅠ), were analyzed by real-time quantitative polymerase (RT-qPCR). Typically, total RNA was extracted using a TRIzol™ reagent (Invitrogen, Carlsbad, CA, USA) and according to the manufacturer’s instructions. The isolated RNA was reverse-transcribed into cDNA using a PrimeScript 1st Strand cDNA Synthesis Kit (Takara, Kyoto, Japan). Afterwards, the expression of osteogenesis-related genes was measured on a Bio-Rad MyiQ single color RT-qPCR system using a SYBR green PCR kit (Takara, Kyoto, Japan). The relative expression levels of target genes were calculated by the 2^−ΔΔCT^ formula and normalized to that of the housekeeping gene glyceraldehyde 3-phosphate dehydrogenase (GAPDH). Each group had three parallel samples. The primers used for RT-qPCR are listed in Table 1.

### 2.8. 3D Bioprinting of Cell-Laden Scaffolds

The inks prepared above were further used to prepare bio-inks by mixing with the cell suspension. The cell density in bio-inks was 1 × 10^7^ cells/mL. In order to improve the biological activity of the scaffold, BMP-2 and PDGF were added into the bio-inks with a final concentration of 5 ug/mL and 100 ng/mL, respectively. Before printing, the working area was first sterilized by UV light irradiation. A nozzle with an inner diameter of 400 μm was used, and the bioprinting process was performed after adjusting the appropriate printing parameters. After printing, the scaffold was transferred to a 4% CaCl_2_ solution, cross-linked for 5 min, and then irradiated for 30 s under 365 nm UV light. Then, the scaffolds were washed with PBS containing 2% penicillin–streptomycin three times and finally replaced with fresh culture medium. Three groups of cell-laden scaffolds were prepared, and according to the different growth factors in the bio-inks, they were labeled as follows: mBMSCs group, mBMSCs/BMP2 group and mBMSCs/PDGF group.

### 2.9. Cell Viability, Proliferation and Differentiation

The cell-laden scaffolds were incubated in culture medium, and the medium was changed every two days. The scaffolds used for cell viability assay were printed in a dimension of 10 mm × 10 mm with two layers. After culturing for 1 and 3 days, cell viability in the cell-laden scaffolds was assessed using a Live-Death Staining Kit according to the manufacturer’s instructions. The live/dead cells were observed by a confocal microscope (Leica, TCSSP8, Germany). At 1, 3 and 5 days, cell proliferation in the cell-laden scaffolds (10 mm × 10 mm, 4 layers) was assessed by CCK8. The absorbance values were measured at 450 nm, with three parallel samples.

For ALP activity assay, the cell-laden scaffolds (10 mm × 10 mm, 4 layers) were printed. After culturing for 3, 7 and 10 days, the scaffolds were crushed evenly; then, 200 μL of 1% Triton X-100 was added to each well and lysed at 4 °C for 30 min. After centrifugation, the supernatant was taken for ALP quantification, and the total protein amount was calculated according to the methods in Section 2.6.1.

For RT-qPCR analysis, the cell-laden scaffolds (10 mm × 10 mm, 10 layers) were printed and cultured in a 12-well plate. At days 7 and 14, the scaffolds were crushed, and the total RNA was extracted using the TRIzol method. The expressions of osteogenesis-related genes (OPN, CCN and Runx2) and the key gene (Fibronectin, FN) linked to soft tissue repair were analyzed by RT-qPCR. The steps of reverse transcription and PCR reaction are as specified in Section 2.7.3.

### 2.10. In Vivo Experiment

#### 2.10.1. Surgical Procedure

Three adult male beagles (average weight = 12–19 kg, 12–15 months) were used for the in vivo experiments. All animal experimental protocols were approved by the Animal Research Committee of Guangzhou Longgui Xingke animal farm (XK19011018, date: 3 December 2019) and were in compliance with the instructions of Institutional Animal Care and Use Committee (IACUC) guidelines.

The periodontal defect model was established as follows: general anesthesia was administered to beagle dogs with pentobarbital at a dose of 30 mg/kg, and local infiltration anesthesia of the operative area was achieved with atticaine containing 1:100,000 norepinephrine. After successful anesthesia, periodontal evaluation, including probing depth (PD) and bleeding index (BI), was performed first. Then, in a lateral position, an opener was placed, and a flap procedure was performed in the mandibular premolar region of the beagle dogs. A buccal periodontal defect of 5 mm × 5 mm (width × depth) was made on 1/3 of the root of the third and fourth mandibular premolars on both sides with a dental drill. The scaffolds were divided into four groups: (1) non-implanted scaffold (blank group), (2) GelMA/SA/BGM scaffold (scaffold group), (3) GelMA/SA/BGM scaffold containing BMP2 and PDGF (BMP2/PDGF group), (4) cell-laden GelMA/SA/BGM scaffold containing BMP2 and PDGF (mBMSCs/BMP2/PDGF group); they were then appropriately sized and placed at the defect sites. The wound was alternately washed with normal saline and gentamicin and then reset and sutured. Penicillin (4 × 10^4^ IU/kg) was injected intramuscularly for 3 days to prevent infection.

#### 2.10.2. Micro-CT Assessment of Osteogenic Behavior around Implants

Eight weeks after the operation, periodontal evaluation was performed, including periodontal probing depth (PD) and gingival bleeding index (BI). Then, the beagles were executed with pentobarbital overdose. Bilateral mandibular samples (premolar to first molar area) were collected and fixed in 4% paraformaldehyde at room temperature for 48 h. Samples were fixed on the camera and scanned one by one with Micro-CT (SKYSCAN1172, Bruker, Karlsruhe, Germany). The rate of newborn bone height (Regeneration height per original defect height, RH/ODH), number of the new bone trabecular mass (trabecular number, Tb.N), and proportion of newborn bone volume to total volume (bone volume per total volume, BV/TV) were calculated and analyzed by NRecon and CTAn software (Bruker, Karlsruhe, Germany).

#### 2.10.3. Histological Evaluation and Histomorphometric Measurement

The samples were removed from the fixation solution, washed overnight with tap water, then dehydrated with gradient ethanol, followed by embedding with photocurable resin (T7200 resin embedding agent). After the resin curing was completed, the tissue sections were cut by a hard tissue slicer in the direction of the buccal tongue; the thickness of the sections was 100 μm. After polishing, HE staining and toluidine blue staining were performed according to the manufacturer’s instructions. Finally, the gingiva and new bone regeneration in the periodontal defect area were observed using a microscope (Olympus DP74, Tokyo, Japan). The histomorphological analysis was performed using a modified method, as described in the previous literature [32,33]. Specifically, the ratio of new bone height and new bone area to initial bone defect height and bone defect area were examined. Height measurements were initiated from the bottom of the bone defect, serving as the boundary, and were executed using image J 1.53e software. The quantification of the original bone defect area and regenerated bone area was accomplished by cropping various regions of the micrograph. The total height and area of the newly generated bone in each photomicrograph were determined by the total color pixels of each image. These measurements were subsequently divided by the total area of bone defect or total height pixels to derive the fraction.

### 2.11. Statistical Analysis

All data were expressed as means ± standard deviations. Statistical analysis was realized using SPSS version 16.0 (Statistical Package, Chicago, USA). One-way ANOVA was performed to discern the differences between groups. A value of *p* < 0.05 was considered to be statistically significant.

## 3. Results

### 3.1. Physicochemical Characterization of 3D-Printed Scaffolds

**Figure 2:**
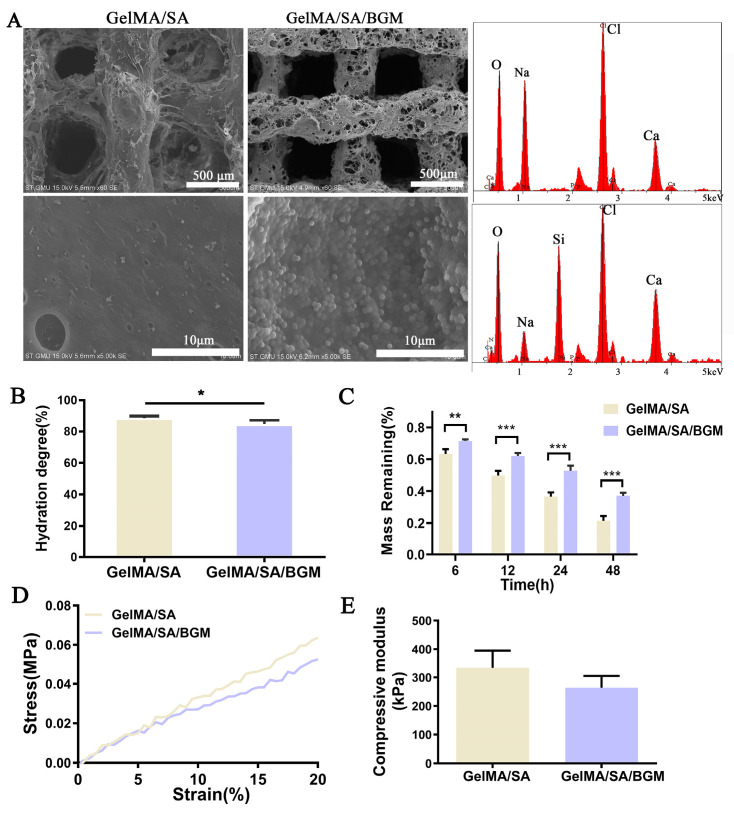
Characterization of GelMA/SA and GelMA/SA/BGM scaffolds. (**A**) SEM images and EDS analysis of GelMA/SA and GelMA/SA/BGM scaffolds. (**B**) Water absorption. (**C**) Degradation rate in the type II collagenase solution. (**D**) Stess-stain curving. (**E**) compressive modulus. * *p* < 0.05, ** *p* < 0.01, *** *p* < 0.001 vs. GelMA/SA.

As shown in Figure 2A, both of the lyophilized scaffolds possessed a connected pore structure along the depositional direction and a similar pore size. A relatively intact filament structure was observed in the GelMA/SA scaffold, while a large number of small pores existed in the filaments of the GelMA/SA/BGM scaffold. At high magnification, the GelMA/SA scaffold showed a relatively smooth surface, while the GelMA/SA/BGM scaffold demonstrated a rough surface, with BGM particles uniformly encapsulated in the matrix. Additionally, the results of EDS analysis demonstrated the increased characteristic peaks of Si, Ca and P in the GelMA/SA/BGM scaffold, indicating that the BGM was successfully embedded in the matrix of the GelMA/SA/BGM scaffold.

The swelling behavior of the scaffolds was tested by immersing them in PBS for 24 h. As shown in Figure 2B, the absorption water content of the GelMA/SA scaffold was significantly higher than that of the GelMA/SA/BGM scaffold. The decrease in water absorption likely resulted from strong interactions between BGM and the GelMA/SA matrix. Both scaffolds showed a rapid degradation rate in the type II collagenase solution. After soaking for 48 h, the degradation of the GelMA/SA scaffold was faster than the GelMA/SA/BGM scaffold, and the mass remaining in the GelMA/SA scaffold was 21.42%, while it was 37.12% for the GelMA/SA/BGM scaffold (Figure 2C).

Figure 2D presents the stress–strain curves of 3D-printed GelMA/SA-based scaffolds. The GelMA/SA/BGM scaffold exhibited a lower deformation than the GelMA/SA scaffold when the strain was greater than 5% and showed a deceased elastic modulus with the addition of BGM (Figure 2E).

**Figure 3:**
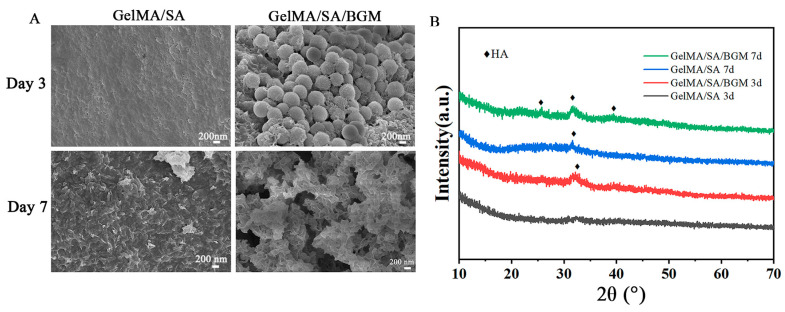
(**A**) SEM images and (**B**) XRD patterns of GelMA/SA and GelMA/SA/BGM after immersing in SBF for 3 and 7 days.

Figure 3A shows the SEM images of the scaffolds after immersion in SBF for 3 and 7 days. At day 3, no obvious mineralized deposits were observed on the surface of the GelMA/SA scaffold, while a small amount of worm-like apatite was deposited on the surface of BGM exposed on the surface of the GelMA/SA/BGM scaffold. After 7 days, a thin layer of needle-like apatite was deposited on the surface of the GelMA/SA scaffold, while the GelMA/SA/BGM scaffold surface was covered by a thick layer of apatite. The XRD patterns demonstrated that only the GelMA/SA/BGM scaffold showed the characteristic peaks of hydroxyapatite at day 3, and when immersed in SBF for 7 days, the characteristic peaks of hydroxyapatite also appeared in the GelMA/SA scaffold (Figure 3B). The in vitro mineralization experiments indicated that incorporating BGM into GelMA/SA hydrogel could improve its bioactivity.

### 3.2. Characterization of Biocompatibility and Osteogenic Properties of 3D-Printed Composite Hydrogel Scaffolds

**Figure 4:**
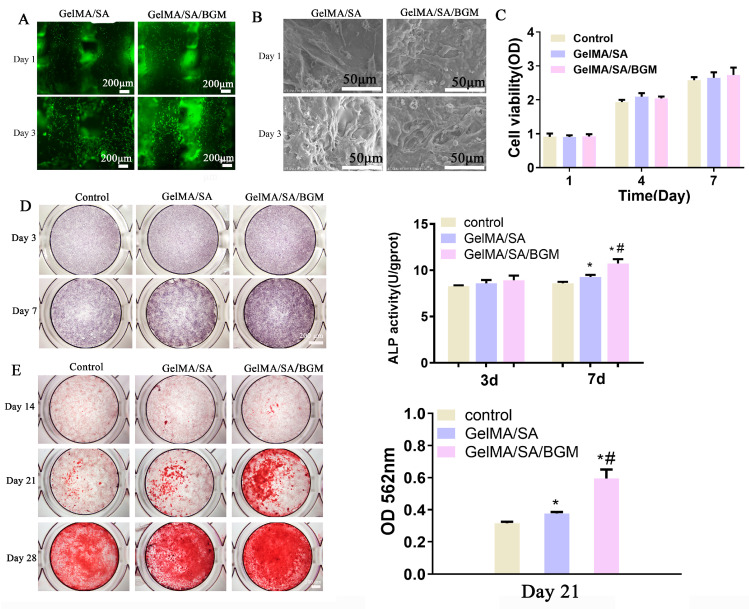
In vitro biocompatibility and osteogenesis evaluation of mBMSCs after culturing with GelMA/SA and GelMA/SA/BGM. (**A**) Live/dead cell staining. (**B**) Cell morphology observation. (**C**) Cell proliferation viability. (**D**) ALP staining and quantitative analysis of mBMSCs incubated with extracts for 3 and 7 days. (**E**) Alizarin red staining and quantitative analysis of mBMSCs incubated with extracts at different time points. * *p* < 0.05 vs. control; ^#^ *p* < 0.05 vs. GelMA/SA.

The viability of mBMSCs on the 3D-printed composite hydrogel scaffolds was evaluated by a live/dead assay. As shown in Figure 4A, live cells (green) adhered to the surfaces of the scaffolds, and no dead cells (red) were observed. Moreover, the number of live cells increased with time. SEM images showed that the cells displayed good spreading morphology and obvious pseudopodia at day 1, and after 3 days, both scaffolds were covered with cells. The cell proliferation assay showed that the OD values of each group increased with time and had no significant difference at days 1, 4 and 7. Regarding the results of live/dead straining, SEM and CCK-8 assays indicated the good biocompatibility of 3D-printed GelMA/SA-based composite scaffolds.

The extract was used to evaluate the capacity of the scaffolds to induce the osteogenic differentiation of mBMSCs by characterizing the ALP activity, calcium nodule deposition and the expression of osteogenic-related genes.

For ALP staining in Figure 4D, all groups appeared to be light purple, and the difference between the groups was not obvious at day 3. After 7 days, the staining of the experimental groups was darker than that of the control group, and the GelMA/SA/BGM group showed the deepest staining, suggesting an enhanced ALP activity of mBMSCs in the presence of BGM. The result of ALP quantitative analysis was consistent with the result of ALP staining.

For alizarin red staining, after culturing for 14 days, only a few mineralized nodules appeared in each group. When cultured for 21 and 28 days, more mineralized nodules were observed in the groups with extracts than the control group, and the GelMA/SA/BGM group had the most mineralized nodules (Figure 4D). The quantification of alizarin red at day 21 demonstrated a significant increase in calcium deposition in the groups with extracts in compassion with the control group; in particular, calcium deposition was significantly enhanced in the group of GelMA/SA/BGM compared with the other two groups (Figure 4E), which was consistent with the staining results. In summary, the 3D-printed GelMA/SA-based composite scaffolds can promote the formation of mineralized nodules and osteogenic differentiation of mBMSCs, and an enhanced osteogenic activity was achieved after the addition of BGM.

**Figure 5:**
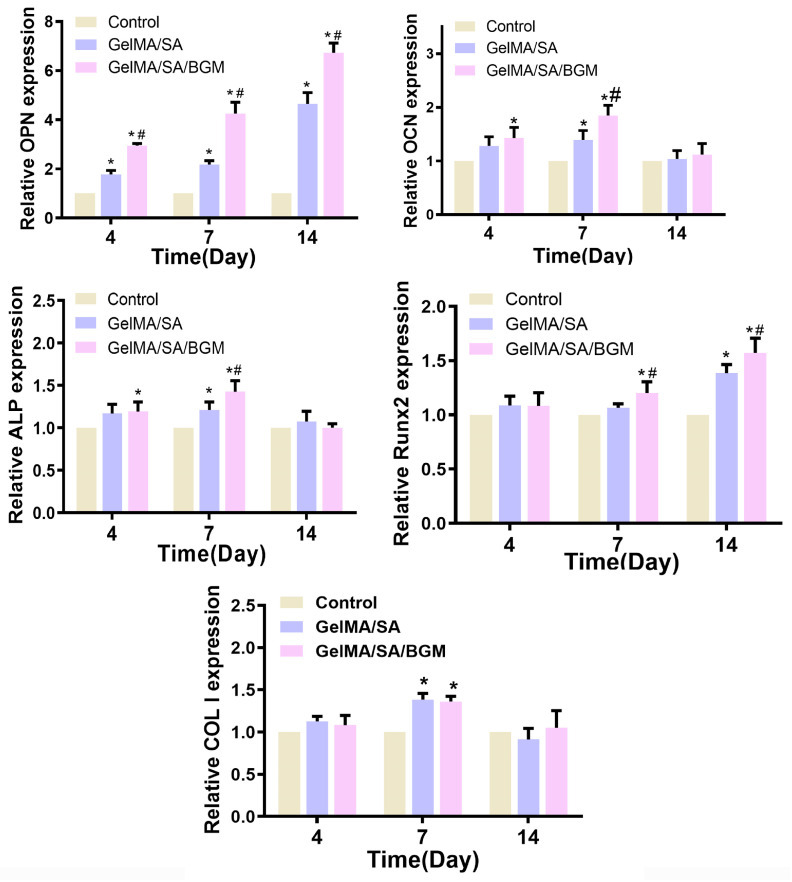
The osteogenic gene expression (ALP, COL1, OCN, OPN and Runx2) of mBMSCs after culturing with extracts for 4, 7 and 14 days. * *p* < 0.05 vs. control; ^#^ *p* < 0.05 vs. GelMA/SA.

To further prove the influence of each scaffold on osteogenic differentiation of mBMSCs, the osteogenic-related genes OPN, OCN, ALP, Runx2 and COL I were analyzed and are shown in Figure 5. At day 4, the expression of OCN, OPN and ALP in the GelMA/SA and GelMA/SA/BGM groups were upregulated compared to the control group, and there was no significant difference for Runx2 and COL I in the three groups. Among them, both the GelMA/SA and GelMA/SA/BGM groups could significantly enhance the expression of OPN. Compared with the control group, only the GelMA/SA/BG group showed a statistical difference in the expression of OCN and ALP. At day 7, compared with the control group, GelMA/SA and GelMA/SA/BGM groups could significantly upregulate the expression of OCN, OPN, Runx2, COL I and ALP, except for the Runx2 expression of GelMA/SA group. At day 14, there was no significant difference in the expression of OCN, ALP and COL I. Compared with the control group, the expression of OPN and Runx2 were significantly upregulated in both GelMA/SA and GelMA/SA/BGM groups, and the gene expression in the GelMA/SA/BGM group was much higher than in the GelMA/SA group. In summary, the effect of the scaffolds in promoting the expression of osteogenesis-related genes was as follows: GelMA/SA/BGM group > GelMA/SA group > control group, in which the GelMA/SA/BGM group showed the best promoting effect.

### 3.3. The Viability, Proliferation, and Related-Gene Expression of Osteogenic Differentiation and Soft Tissue Repair of mBMSCs in 3D-Bioprinted Cell- and Growth Factor-Laden Scaffolds

**Figure 6:**
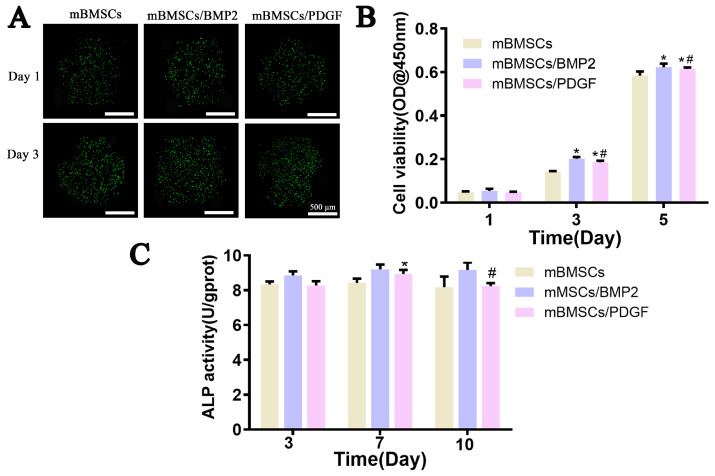
Cell viability, proliferation and ALP activity evaluation of mBMSCs encapsulated in GelMA/SA/BGM hydrogel after 3D bioprinting. (**A**) Live/dead cell staining. (**B**) Cell proliferation. (**C**) ALP activity. * *p* < 0.05 vs. control, ^#^ *p* < 0.05 vs. GelMA/SA.

After 3D bioprinting, the cell- and growth factor-laden scaffolds were incubated in culture medium for 1 and 3 days, and live/dead cell staining was used to evaluate cell viability in 3D-printed scaffolds. The results showed that mBMSCs encapsulated in the scaffolds exhibited good cell vitality, and the number increased over time (Figure 6A), which is also confirmed by the CCK-8 test. The BMP2- and PDGF-loaded scaffolds could considerably increase cell proliferation at days 3 and 5 when compared to the scaffold devoid of growth factors (Figure 6B), indicating that BMP2 and PDGF could promote the proliferation of mBMSCs. Furthermore, the ALP activity of mBMSCs in 3D-bioprinted scaffolds at days 3, 7 and 10 is shown in Figure 6C. There was no significant difference in ALP activity among the three groups after an incubation of 3 days. mBMSCs/BMP2 and mBMSCs/PDGF scaffolds exhibited a significantly enhanced ALP activity at day 7 in comparison to the mBMSCs scaffold, and the cell-laden scaffold with BMP2 showed the highest ALP activity. A significant increase in ALP activity in the mBMSCs/BMP2 scaffold was observed at day 10 when compared to the mBMSCs/PDGF and mBMSCs scaffolds. Therefore, incorporation of BMP2 into the cell-laden scaffold was more conducive to ALP activity expression.

**Figure 7:**
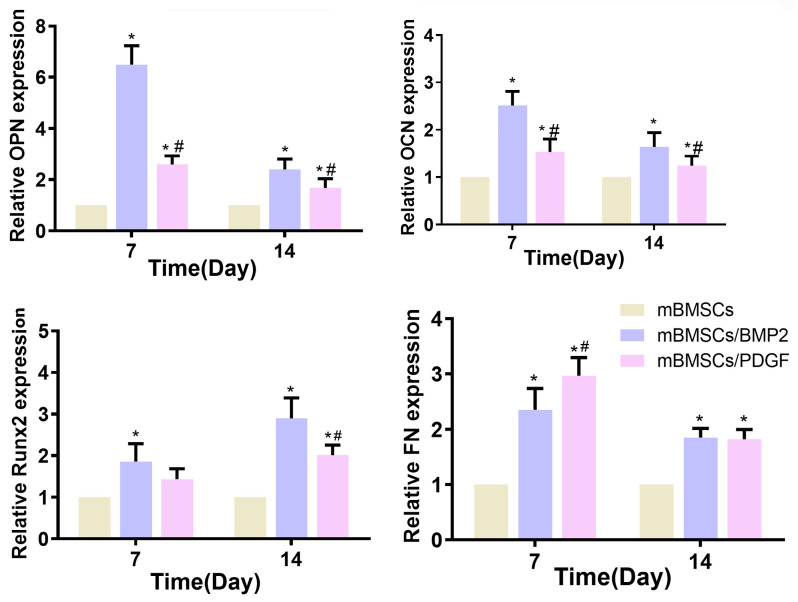
The expression of OPN, OCN, Runx2 and FN of mBMSCs in 3D-bioprinted scaffolds after culturing for 7 and 14 days. * *p* < 0.05 vs. mBMSCs scaffold, ^#^ *p* < 0.05 vs. mBMSCs/BMP scaffold.

The capacity of the 3D-bioprinted scaffolds to promote osteogenic differentiation and soft tissue repair was evaluated by examining the expression of osteogenesis-related (OPN, OCN and Runx2) and soft tissue-related (FN) markers. As shown in Figure 7, compared with the cell-laden scaffold without growth factors (control group), cell- and growth factor-loaded scaffolds could promote the expression of OCN, OPN, Runx2 and FN, and significant differences were observed, except for Runx2 in the mBMSCs/PDGF group at days 7 and 14. The mBMSCs/BMP2 scaffold exhibited the most pronounced expression of genes related to osteogenesis. At day 7, the expression of FN in the mBMSCs/PDGF scaffold was significantly higher than that observed in mBMSCs/BMP2 and mBMSCs scaffolds; the expression of FN in the mBMSCs/PDGF and mBMSCs/BMP scaffolds was comparable, and both significantly differed for the control group at day 14. In summary, the mBMSCs/BMP2 scaffold and mBMSCs/PDGF scaffold could significantly promote the expression of genes related to osteogenesis soft tissue, respectively.

### 3.4. In Vivo Periodontal Repair Experiment

**Figure 8:**
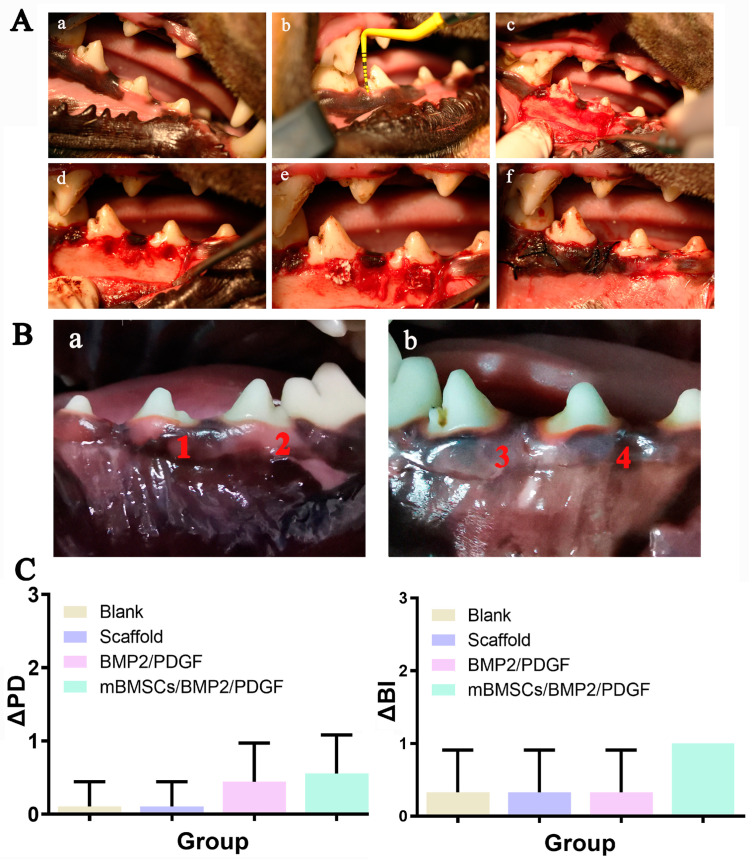
(**A**) Construction of acute periodontal defect in beagle dogs, (a) preoperative photograph, (b) periodontal examination, (c) flap, (d) construction of 5 mm × 5 mm bone defect, (e) scaffold implantation, (f) suture. (**B**) Intraoral photographs at 8 weeks after surgery. (a): (1) blank group, (2) scaffold group; (b): (3) BMP2/PDGF group, (4) mBMSCs/BMP2/PDGF group. (**C**) Periodontal status at 8 weeks after surgery.

In order to examine the periodontal regeneration potential of 3D-bioprinted composite hydrogel scaffolds in vivo, three distinct groups of scaffolds were utilized, namely the GelMA/SA/BGM scaffold group (scaffold group), the GelMA/SA/BGM scaffold with BMP2 and PDGF group (BMP2/PDGF group) and the GelMA/SA/BGM scaffold with BMP2, PDGF and mBMSCs group (mBMSCs/BMP2/PDGF group). These scaffolds were implanted into the periodontal defect model of beagle dogs, while the blank control group (blank group) received no treatment. The surgical procedures involved in the creation of the periodontal defect model and scaffold implantation are illustrated in Figure 8A. After 8 weeks of implantation, all beagle dogs were in good condition without infection, necrosis or wound exposure (Figure 8B). The results of periodontal examination showed an increase in ΔPD value in BMP2/PDGF and mBMSCs/BMP2/PDGF groups as compared with the blank group and scaffold group, with no significant difference (Figure 8C). These findings suggest that the 3D-bioprinted cell-laden scaffold containing BMP2 and PDGF may facilitate the healing of gingival tissue to some extent.

**Figure 9:**
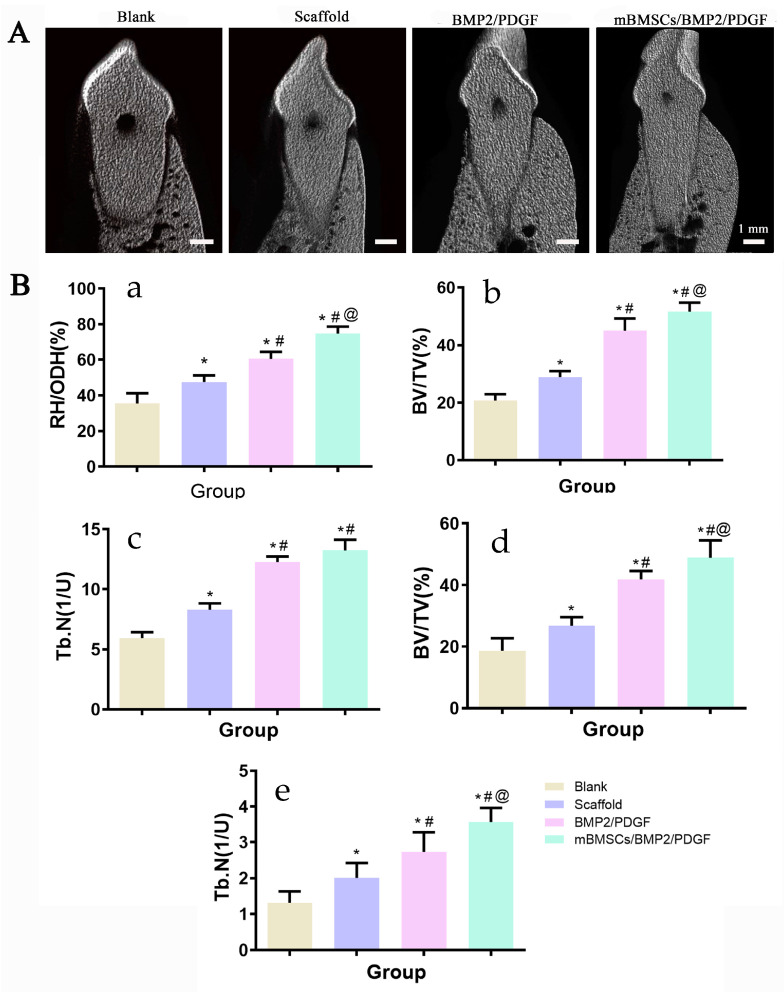
(**A**) Buccal-lingual micro-CT reconstruction 8 weeks after surgery. (**B**) Micro-CT analysis: (a) RH/ODH, (b) 2D analysis of BV/TV, (c) 2D analysis of Tb.N, (d) 3D analysis of BV/TV, (e) 3D analysis of Tb.N. * *p* < 0.05 vs. blank group, ^#^ *p* < 0.05 vs. scaffold group, ^@^ *p* < 0.05 vs. BMP2/PDGF group.

The regeneration of the buccal alveolar bone plate was evaluated using the buccolingual three-dimensional reconstruction of the periodontal model using CTvox software (Bruker, Karlsruhe, Germany). After being treated with 3D-bioprinted scaffolds, the experiment groups exhibited obvious newly formed bone, as depicted in Figure 9A. In addition, the RH/ODH, Tb.N and BV/TV of the experiment groups on one-, two- and three-dimensional scales significantly increased when compared with the blank group, with the mBMSCs/BMP2/PDGF group exhibiting the highest increase, followed by the BMP/PDGF group, scaffold group and control group. The results showed that mBMSCs and growth factor-laden 3D-bioprinted scaffolds can obviously promote the reconstruction of periodontal tissue.

**Figure 10:**
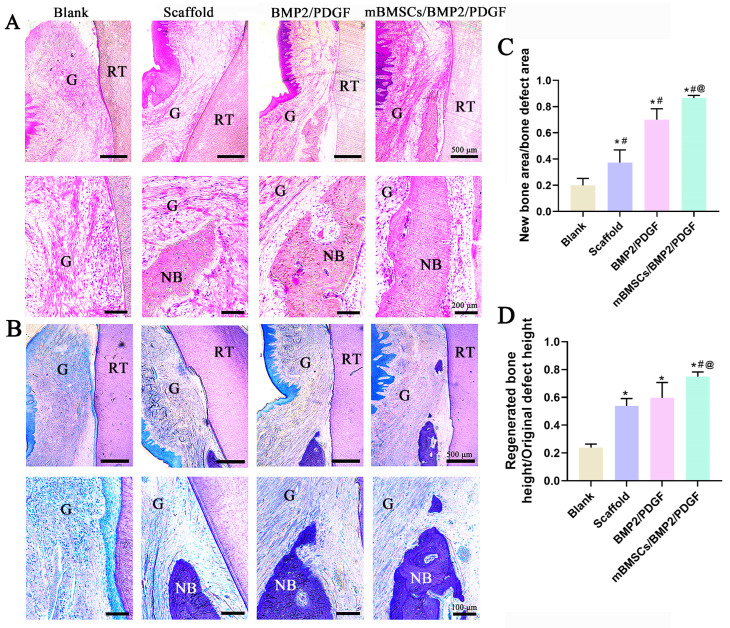
Histological evaluation and histomorphometric analysis of the periodontal tissue regeneration at 8 weeks after surgery. (**A**) H&E staining. (**B**) TB staining. (NB: new bone; RT: root; G: gingiva). (**C**) Ratio of new bone area to total bone defect area. (**D**) Ratio of new bone height to total bone defect height. * *p* < 0.05 vs. blank group, ^#^ *p* < 0.05 vs. scaffold group, ^@^ *p* < 0.05 vs. BMP2/PDGF group.

HE staining results indicated that the scaffolds of all the experimental groups were completely decomposed, and newly formed gingiva and bone tissue were observed in all the experimental groups. In contrast, the blank group was characterized by the hyperplasia of fibrous connective tissue. Notably, the mBMSCs/BMP2/PDGF group exhibited the best effect on the repair of gum and bone tissue as compared to the other groups (Figure 10A). The results of toluidine blue staining were consistent with those of HE (Figure 10B). Compared with the blank group, the area and height of the new bone in the experimental groups were significantly improved, and the repair effect of the scaffold containing cells and growth factors was more obvious (Figure 10C,D). These results further confirmed the potential of 3D-bioprinted composite scaffolds with cells and growth factors to regenerate periodontal tissue.

## 4. Discussion

The ideal periodontal repair should involve restoring both soft and hard periodontal tissues. Although tissue engineering techniques exhibit potential in regenerating periodontal tissues, the lack of appropriate scaffold materials has hindered the attainment of this objective. Nevertheless, recent advancements in 3D printing technology have facilitated the production of scaffolds with complex structures. This technique not only enables personalized restorations but also simulates the microenvironment of tissues and organs, providing an optimal environment for cell growth and extracellular matrix formation [34,35,36]. With the development of 3D printing technology and biomaterials, 3D bioprinting of cell-laden composite hydrogel scaffolds has become a new direction for periodontal tissue regeneration. This novel biomanufacturing technology enables the precise construction of cells and cell encapsulation materials [37]. To achieve successful 3D bioprinting, bio-inks aimed at preserving the cellular phenotype and function are required.

GelMA, a hydrogel material synthesized through chemical polymerization of gelatin and MA, exhibits a structure similar to natural ECM and possesses favorable biocompatibility and biodegradability [38]. SA is a commonly employed hydrogel material in bioprinting due to its high viscosity and relatively simple cross-linking mechanism [39,40]. However, the surface structure of SA is not conducive to cell adhesion, necessitating the incorporation of other biomolecules to enhance cell adhesion [10]. Although the composite hydrogel consisting of GelMA and SA exhibits favorable biocompatibility and printability, its application in bone tissue engineering remains restricted due to the absence of biological activity. To enhance the bioactivity of the composite hydrogel, BGM is commonly employed as a bioactive agent, owing to its unique biological properties, particularly its capacity to stimulate osteogenesis and angiogenesis [41]. In this study, a multi-component hydrogel was successfully synthesized by combining inorganic bioactive particles (BGM) and a polymer matrix (GelMA and SA), which was then processed into GelMA/SA/BGM composite hydrogel scaffolds by 3D printing technology. The physicochemical characteristics and in vitro osteogenic ability of the scaffolds were evaluated. Subsequently, the GelMA/SA/BGM composite hydrogel was explored as a bio-ink for the fabrication of cell and growth factor (BMP2 and PDGF)-laden scaffolds using 3D bioprinting for the regeneration of periodontal tissue.

The regular arrangement of pore structure with similar pore size was observed in GelMA/SA and GelMA/SA/BGM 3D-printed scaffolds. Elemental analysis revealed the presence of Ca, Si and P in the GelMA/SA/BGM scaffold due to the addition of BGM particles, while the GelMA/SA scaffold also contained Ca, potentially resulting from the cross-linking of CaCl_2_ with SA. Both GelMA/SA and GelMA/SA/BGM scaffolds exhibited high water content, facilitating nutrient and metabolite flow and transportation, and creating a favorable microenvironment for tissue regeneration and repair. The GelMA/SA/BGM scaffold exhibited a slightly lower water content compared to the GelMA/SA scaffold. This can be attributed to the potential interaction between the Ca ions dissolved from BGM and the carboxyl groups of GelMA, leading to a decrease in the binding capacity of the GelMA and water molecules [28]. Additionally, the uniform dispersion of BGM within the scaffolds resulted in a decreased binding rate of water molecules to GelMA and SA, ultimately contributing to the reduction in water content. The degradation rate of the two scaffolds in the presence of type II collagenase exhibited an initial rapid decline followed by a gradual decrease, with the scaffold containing BGM demonstrating a slower degradation rate. This phenomenon may be attributed to the superior water absorption capacity of the GelMA/SA scaffold compared to the GelMA/SA/BGM scaffold, which facilitated rapid adsorption of the type II collagenase solution and subsequent degradation. Consequently, the incorporation of BGM into the GelMA/SA scaffold resulted in a marked improvement in the stability of the hydrogel system. The mechanical testing results indicated a reduction in compression modulus upon the inclusion of BGM, attributable to the presence of BGM impeding the interaction of GelMA molecules activated by PI-2959. The ability to induce apatite formation in vitro may indirectly reflect the in vivo osseointegration ability of biomaterials [42]. The biomineralization assay conducted in vitro demonstrated the formation of a layer of apatite on the GelMA/SA/BGM scaffold surface, signifying the favorable bioactivity of this composite material system, suitable for the fabrication of bone tissue engineering scaffolds. The GelMA/SA-based scaffolds exhibited good biocompatibility and cell viability. The incorporation of BGM into the scaffold resulted in enhanced cell adhesion, proliferation, ALP activity, calcium nodule formation and the promotion of osteogenesis-related gene expression. The improvement of the biological performance of the BGM-loaded scaffold can be attributed to the bioactive ions (Si, Ca and P) released from BGM, which have been shown to promote the osteogenesis and angiogenesis of mesenchymal stem cells in many studies [43,44].

Based on the above experimental results, the composite hydrogel composed of GelMA, SA and BGM presented great potential as a bio-ink for 3D bioprinting of cell-laden scaffolds. Both BMP2 and PDGF are growth factors that are associated with regenerative repair of maxillofacial tissues, and they are capable of recruiting mesenchymal stem cells (MSCs). BMP2 primarily induces bone regeneration by promoting the differentiation of MSCs into osteoblasts [45,46]. On the other hand, PDGF is a potent chemotactic agent that can attract MSCs, fibroblasts, repair proteins, and other factors, thereby promoting soft tissue regeneration [47]. Consequently, we developed a scaffold laden with mBMSCs, BMP2, and PDGF via 3D bioprinting to address the repair of the defects resulting from periodontal disease. The utilization of PDGF-B facilitates the restoration of gingival tissue, whereas BMP2 expedites the generation of fresh osseous tissue. Our investigation involved in vitro and in vivo experiments to evaluate the scaffold’s efficacy for bone and soft tissue repair, with potential applications for personalized tissue defect therapy.

Natural polymer hydrogels, such as GelMA and SA, exhibit favorable biocompatibility and low cytotoxicity, rendering them suitable carriers for delivering diverse biomolecules, including living cells [48]. Bioactive glass is classified as a type of bioactive material that possesses favorable biocompatibility and osteoinductivity [41]. Therefore, the 3D bioprinted GelMA/SA/BGM scaffolds with cells and growth factors exhibited favorable cell viability and proliferation activity. The ALP activity of the scaffold was enhanced after the addition of BMP2. OCN, OPN and Runx2 are markers for different stages of bone matrix mineralization and osteogenic differentiation. FN, as one of the components of the extracellular matrix, promotes fibroblast migration [49] and is thus considered a marker for soft tissue repair. RT-qPCR results showed that the mBMSCs/BMP2 scaffold exhibited a greater capacity to enhance the expression of OCN, OPN and Runx2 in mBMSCs, while the mBMSCs/PDGF scaffold demonstrated a superior ability to promote the expression of FN in mBMSCs. Additionally, the expression of OCN, OPN and FN was observed to be more pronounced during the initial stage, while Runx2 exhibited greater activity during later stages.

In vivo, we constructed the mBMSCs/BMP2/PDGF scaffold by combining the mBMSCs/PDGF scaffold, which promotes soft tissue repair, and the mBMSCs/BMP2 scaffold, which promotes bone regeneration. The results indicated that postoperative wound healing was satisfactory, with improvements in ΔPD and ΔSI. The mBMSCs/BMP2/PDGF scaffold exhibited the most significant improvement, providing evidence that the mBMSCs/BMP2/PDGF scaffold is an effective means of promoting gingival repair. Additionally, new bone tissue was observed in all groups, with the mMSCs/BMP2/PDGF group exhibiting the most significant improvement in buccal alveolar bone height, bone tissue volume, and bone trabecular number, confirming that adding BMP2 in cell-laden scaffolds can effectively promote bone formation. Thus, it can be concluded that cell-laden scaffolds in combination with BMP2 and PDGF can effectively repair periodontal soft and hard tissue defects when compared with the scaffold only containing BMP2 and PDGF.

Although we successfully established a bio-ink that can be used for 3D bioprinting and achieved the desired bone and soft tissue regeneration in an in vivo periodontal defect experiment, the research still has some shortcomings. The low mechanical strength of the scaffold should be further improved to be more suitable for the repair of periodontal bone tissue defects. Additionally, an acute trauma model of periodontal tissue was used in this study, and the repair of periodontal injury caused by periodontal inflammation and the regulation effect of hydrogel on inflammation should be carried out in future studies.

## 5. Conclusions

In this study, we prepared a multi-component hydrogel composed of GelMA, SA and BGM and fabricated it into a porous scaffold by extrusion-based 3D printing technology; due to its good biocompatibility and osteogenic activity, the composite hydrogel can be used as an alternative material to encapsulate cells and growth factors for 3D bioprinting. The hydrogel’s capacity to be printed was unaffected by the inclusion of cells or growth factors, and the 3D-bioprinted hydrogel scaffolds were able to mimic the biological effects of the loaded growth factors. BMP2 and PDGF loaded in the scaffolds may help mBMSCs differentiate into osteoblasts and improve their capacity to heal soft tissue, respectively. The dual functional scaffold showed a good potential for periodontal tissue regeneration, which can facilitate the restoration of the alveolar bone and the healing of gingival tissue in the periodontal defect model of beagle dogs.

Therefore, this study provides a promising bio-ink for preparing cell-laden 3D bioprinted scaffolds, and different functional biological factors such as BMP2 or PDGF can be incorporated into the bio-ink that may have versatile applications in the individualized therapy of tissue defects.

## Figures and Tables

**Table 1:** Prime sequences.

Gene	Primer Sequences
ALP	Forward: 5′-TGCCTACTTGTGTGGCGTGAA-3′ Reverse: 5′-TCACCCGAGTGGTAGTCACAATG-3′
OPN	Forward: 5′-TCCAAAGCCAGCCTGGAAC-3′ Reverse: 5′-TGACCTCAGAAGATGAACTC-3′
OCN	Forward: 5′-GCTTGTGACGAGCTATCAGACCAG-3′ Reverse: 5′-AGCTGCTGTGACATCCATACTTGC-3′
Runx2	Forward: 5′-TCAGCGTCAACACCATCATTC-3′ Reverse: 5′-CCAGACCAGCAGCACTCCATA-3′
COL I	Forward: 5′-AGAACAGCGTGGCCT-3′ Reverse: 5′-TCCGGTGTGACTCGT-3′
FN	Forward: 5′-ATGTGGACCCCTCCTGATAGT-3′ Reverse: 5′-GCCCAGTGATTTCAGCAAAGG-3′
GAPDH	Forward: 5′-TGTGTCCGTCGTGGATCTG-3′ Reverse: 5′-TTGCTGTTGAAGTCGCAGGA-3′

## Data Availability

All relevant data of this study are presented. Additional data will be provided upon request.

## References

[1] Zhang L., Dong Y., Xue Y., Shi J., Zhang X., Liu Y., Midgley A.C., Wang S. (2019). Multifunctional triple-layered composite scaffolds combining platelet-rich fibrin promote bone regeneration. ACS Biomater. Sci. Eng..

[2] Jamuna-Thevi K., Saarani N.N., Kadir M.R.A., Hermawan H. (2014). Triple-layered PLGA/nanoapatite/lauric acid graded composite membrane for periodontal guided bone regeneration. Mater. Sci. Eng. C.

[3] Sowmya S., Mony U., Jayachandran P., Reshma S., Kumar R.A., Arzate H., Nair S.V., Jayakumar R. (2017). Tri-Layered Nanocomposite Hydrogel Scaffold for the Concurrent Regeneration of Cementum, Periodontal Ligament, and Alveolar Bone. Adv. Heal. Mater..

[4] Pilipchuk S.P., Fretwurst T., Yu N., Larsson L., Kavanagh N., Asa’Ad F., Cheng K.C.K., Lahann J., Giannobile W.V. (2018). Micropatterned scaffolds with immobilized growth factor genes regenerate bone and periodontal ligament-like tissues. Adv. Health Mater..

[5] Soltani-Dehnavi S., Mehdikhani M., Rafienia M., Bonakdar S. (2018). Preparation and in vitro evaluation of polycaprolactone/PEG/bioactive glass nanopowders nanocomposite membranes for GTR/GBR applications. Mater. Sci. Eng. C.

[6] Asa’Ad F., Pagni G., Pilipchuk S.P., Giannì A.B., Giannobile W.V., Rasperini G. (2016). 3D-printed scaffolds and biomaterials: Review of alveolar bone augmentation and periodontal regeneration applications. Int. J. Dent..

[7] Dubey N., Ferreira J.A., Daghrery A., Aytac Z., Malda J., Bhaduri S.B., Bottino M.C. (2020). Highly tunable bioactive fiber-reinforced hydrogel for guided bone regeneration. Acta Biomater..

[8] Wang C., Huang W., Zhou Y., He L., He Z., Chen Z., He X., Tian S., Liao J., Lu B. (2020). 3D printing of bone tissue engineering scaffolds. Bioact. Mater..

[9] Murphy S.V., Atala A. (2014). 3D bioprinting of tissues and organs. Nat. Biotechnol..

[10] Wu J., Miao G., Zheng Z., Li Z., Ren W., Wu C., Li Y., Huang Z., Yang L., Guo L. (2019). 3D printing mesoporous bioactive glass/sodium alginate/gelatin sustained release scaffolds for bone repair. J. Biomater. Appl..

[11] Tasnim N., De la Vega L., Anil Kumar S., Abelseth L., Alonzo M., Amereh M., Joddar B., Willerth S.M. (2018). 3D bioprinting stem cell derived tissues. Cell. Mol. Bioeng..

[12] Aljohani W., Ullah M.W., Zhang X., Yang G. (2017). Bioprinting and its applications in tissue engineering and regenerative medicine. Int. J. Biol. Macromol..

[13] Yang X., Ma Y., Wang X., Yuan S., Huo F., Yi G., Zhang J., Yang B., Tian W. (2023). A 3D-bioprinted functional module based on decellularized extracellular matrix bioink for periodontal regeneration. Adv. Sci..

[14] Shaikh M.S., Zafar M.S., Alnazzawi A., Javed F. (2022). Nanocrystalline hydroxyapatite in regeneration of periodontal intrabony defects: A systematic review and meta-analysis. Ann. Anat..

[15] Shaikh M.S., Husain S., Lone M.A., Lone M.A., Akhlaq H., Zafar M.S. (2020). Clinical effectiveness of anorganic bovine-derived hydroxyapatite matrix/cell-binding peptide grafts for regeneration of periodontal defects: A systematic review and meta-analysis. Regen. Med..

[16] Zhang H., Wu S., Chen W., Hu Y., Geng Z., Su J. (2023). Bone/cartilage targeted hydrogel: Strategies and applications. Bioact. Mater..

[17] Correa S., Grosskopf A.K., Hernandez H.L., Chan D., Yu A.C., Stapleton L.M., Appel E.A. (2021). Translational applications of hydrogels. Chem. Rev..

[18] Zhang R., Xie L., Wu H., Yang T., Zhang Q., Tian Y., Liu Y., Han X., Guo W., He M. (2020). Alginate/laponite hydrogel microspheres co-encapsulating dental pulp stem cells and VEGF for endodontic regeneration. Acta Biomater..

[19] Zhao Z., Wang Z., Li G., Cai Z., Wu J., Wang L., Deng L., Cai M., Cui W. (2021). Injectable microfluidic hydrogel microspheres for cell and drug delivery. Adv. Funct. Mater..

[20] Huang Q., Zou Y., Arno M.C., Chen S., Wang T., Gao J., Dove A.P., Du J. (2017). Hydrogel scaffolds for differentiation of adipose-derived stem cells. Chem. Soc. Rev..

[21] Yang J., Yang K., Man W., Zheng J., Cao Z., Yang C.-Y., Kim K., Yang S., Hou Z., Wang G. (2023). 3D bio-printed living nerve-like fibers refine the ecological niche for long-distance spinal cord injury regeneration. Bioact. Mater..

[22] Ma Y., Yang X., Chen Y., Zhang J., Gai K., Chen J., Huo F., Guo Q., Guo W., Gou M. (2023). Biomimetic peridontium patches for functional periodontal regeneration. Adv. Healthc. Mater..

[23] Fang Y., Guo Y., Ji M., Li B., Guo Y., Zhu J., Zhang T., Xiong Z. (2022). 3D printing of cell-laden microgel-based biphasic bioink with heterogeneous microenvironment for biomedical applications. Adv. Funct. Mater..

[24] Wang J., Wang C., Wang Q., Zhang Z., Wang H., Wang S., Chi Z., Shang L., Wang W., Shu Y. (2022). Microfluidic preparation of gelatin methacryloyl microgels as local drug delivery vehicles for hearing loss therapy. Acs Appl. Mater. Inter..

[25] Bendtsen S.T., Quinnell S.P., Wei M. (2017). Development of a novel alginate-polyvinyl alcohol-hydroxyapatite hydrogel for 3D bioprinting bone tissue engineered scaffolds. J. Biomed. Mater. Res. A.

[26] Gao F., Xu Z., Liang Q., Liu B., Li H., Wu Y., Zhang Y., Lin Z., Wu M., Ruan C. (2018). Direct 3D printing of high strength biohybrid gradient hydrogel scaffolds for efficient repair of osteochondral defect. Adv. Funct. Mater..

[27] Gao Q., Niu X., Shao L., Zhou L., He Y. (2019). 3D printing of complex GelMA-based scaffolds with nanoclay. Biofabrication.

[28] Zheng J., Zhao F., Zhang W., Mo Y., Zeng L., Li X., Chen X. (2018). Sequentially-crosslinked biomimetic bioactive glass/gelatin methacryloyl composites hydrogels for bone regeneration. Mater. Sci. Eng. C.

[29] Tian T., Xie W., Gao W., Wang G., Zeng L., Miao G., Lei B., Lin Z., Chen X. (2019). Micro-nano bioactive glass particles incorporated porous scaffold for promoting osteogenesis and angiogenesis in vitro. Front. Chem..

[30] Hu Q., Chen X.F., Zhao N.R., Li Y.L., Mao C. (2014). Fabrication and characterization of dodecylamine derived monodispersed mesoporous bioactive glass sub-micron spheres. J. Sol-Gel Sci. Technol..

[31] Van Den Bulcke A.I., Bogdanov B., De Rooze N., Schacht E.H., Cornelissen M., Berghmans H. (2000). Structural and rheological properties of methacrylamide modified gelatin hydrogels. Biomacromolecules.

[32] Emerton K., Drapeau S., Prasad H., Rohrer M., Roffe P., Hopper K., Schoolfield J., Jones A., Cochran D. (2011). Regeneration of periodontal tissues in non-human primates with rhGDF-5 and beta-tricalcium phosphate. J. Dent. Res..

[33] Zhang Y., Miron R.J., Li S., Shi B., Sculean A., Cheng X. (2015). Novel mesoporous bioglass/silk scaffold containing adPDGF-B and adBMP7 for the repair of periodontal defects in beagle dogs. J. Clin. Periodontol..

[34] Jian Z., Zhuang T., Qinyu T., Liqing P., Kun L., Xujiang L., Diaodiao W., Zhen Y., Shuangpeng J., Xiang S. (2021). 3D bioprinting of a biomimetic meniscal scaffold for application in tissue engineering. Bioact. Mater..

[35] Li T., Zhai D., Ma B., Xue J., Zhao P., Chang J., Gelinsky M., Wu C. (2019). 3D printing of hot dog-like biomaterials with hierarchical architecture and distinct bioactivity. Adv. Sci..

[36] Zhou G., Jiang H., Yin Z., Liu Y., Zhang Q., Zhang C., Pan B., Zhou J., Zhou X., Sun H. (2018). In vitro regeneration of patient-specific ear-shaped cartilage and its first clinical application for auricular reconstruction. EBioMedicine.

[37] Gu Q., Hao J., Lu Y., Wang L., Wallace G.G., Zhou Q. (2015). Three-dimensional bio-printing. Sci. China Life Sci..

[38] Nichol J.W., Koshy S.T., Bae H., Hwang C.M., Yamanlar S., Khademhosseini A. (2010). Cell-laden microengineered gelatin methacrylate hydrogels. Biomaterials.

[39] Axpe E., Oyen M. (2016). Applications of alginate-based bioinks in 3D bioprinting. Int. J. Mol. Sci..

[40] Ooi H.W., Mota C., ten Cate A.T., Calore A., Moroni L., Baker M.B. (2018). Thiol–ene alginate hydrogels as versatile bioinks for bioprinting. Biomacromolecules.

[41] Zheng K., Sui B., Ilyas K., Boccaccini A.R. (2021). Porous bioactive glass micro- and nanospheres with controlled morphology: Developments, properties and emerging biomedical applications. Mater. Horiz..

[42] Kokubo T., Takadama H. (2006). How useful is SBF in predicting in vivo bone bioactivity?. Biomaterials.

[43] Hoppe A., Güldal N.S., Boccaccini A.R. (2011). A review of the biological response to ionic dissolution products from bioactive glasses and glass-ceramics. Biomaterials.

[44] Mao L., Xia L., Chang J., Liu J., Jiang L., Wu C., Fang B. (2017). The synergistic effects of Sr and Si bioactive ions on osteogenesis, osteoclastogenesis and angiogenesis for osteoporotic bone regeneration. Acta Biomater..

[45] Lissenberg-Thunnissen S.N., de Gorter D.J.J., Sier C.F.M., Schipper I.B. (2011). Use and efficacy of bone morphogenetic proteins in fracture healing. Int. Orthop..

[46] Miron R.J., Zhang Y.F. (2012). Osteoinduction: A review of old concepts with new standards. J. Dent. Res..

[47] Sarment D.P., Cooke J.W., Miller S.E., Jin Q., McGuire M.K., Kao R.T., McClain P.K., McAllister B.S., Lynch S.E., Giannobile W.V. (2006). Effect of rhPDGF-BB on bone turnover during periodontal repair. J. Clin. Periodontol..

[48] Ansari S., Sarrion P., Hasani-Sadrabadi M.M., Aghaloo T., Wu B.M., Moshaverinia A. (2017). Regulation of the fate of dental-derived mesenchymal stem cells using engineered alginate-GelMA hydrogels. J. Biomed. Mater. Res. A.

[49] Den Braber E.T., De Ruijter J.E., Ginsel L.A., Von Recum A.F., Jansen J.A. (1998). Orientation of ECM protein deposition, fibroblast cytoskeleton, and attachment complex components on silicone microgrooved surfaces. J. Biomed. Mater. Res..

